# Woody Plant Transformation: Current Status, Challenges, and Future Perspectives †

**DOI:** 10.3390/plants14223420

**Published:** 2025-11-08

**Authors:** Bal Krishna Maharjan, Md Torikul Islam, Adnan Muzaffar, Timothy J. Tschaplinski, Gerald A. Tuskan, Jin-Gui Chen, Xiaohan Yang

**Affiliations:** 1 Oak Ridge National Laboratory, Biosciences Division, Oak Ridge, TN 37831, USA; maharjanbk@ornl.gov (B.K.M.); islamt@ornl.gov (M.T.I.); muzaffara@ornl.gov (A.M.); tschaplinstj@ornl.gov (T.J.T.); tuskanga@ornl.gov (G.A.T.); 2 The Center for Bioenergy Innovation, Oak Ridge National Laboratory, Oak Ridge, TN 37831, USA

**Keywords:** *Agrobacterium tumefaciens*, *Rhizobium rhizogenes*, hairy root, developmental regulators, in planta transformation

## Abstract

Woody plants, comprising forest and fruit tree species, provide essential ecological and economic benefits to society. Their genetic improvement is challenging due to long generation intervals and high heterozygosity. Genetic transformation, which combines targeted DNA delivery with plant regeneration from transformed cells, offers a powerful alternative to accelerating their domestication and improvement. *Agrobacterium tumefaciens*, *Rhizobium rhizogenes*, and particle bombardment have been widely used for DNA delivery into a wide variety of explants, including leaves, stems, hypocotyls, roots, and embryos, with regeneration occurring via direct organogenesis, callus-mediated organogenesis, somatic embryogenesis, or hairy root formation. Despite successes, conventional approaches are hampered by low efficiency, genotype dependency, and a reliance on challenging tissue culture. This review provides a critical analysis of the current landscape in woody plant transformation, moving beyond a simple summary of techniques to evaluate the co-evolution of established platforms with disruptive technologies. Key advances among these include the use of developmental regulators to engineer regeneration, the rise in in planta systems to bypass tissue culture, and the imperative for DNA-free genome editing to meet regulatory and public expectations. By examining species-specific breakthroughs in key genera, including *Populus*, *Malus*, *Citrus*, and *Pinus*, this review highlights a paradigm shift from empirical optimization towards rational, predictable engineering of woody plants for a sustainable future.

## 1. Introduction

Woody plants are vital sources of energy, building materials, and food, while also providing services such as carbon storage, biodiversity habitat, and climate regulation [1]. Representing approximately 45% of all terrestrial plant species, they range from non-domesticated forest tree species to highly domesticated fruit tree cultivars [2]. Despite their importance, all woody plants face escalating threats from pests, diseases, and environmental extremes. Conventional breeding has been the primary tool for their improvement, but progress has been inherently slow due to long juvenility periods, high heterozygosity, and the vast resources required to manage large breeding populations [3]. Genetic transformation and the recent advent of genome editing technologies offer transformative potential to accelerate the development of woody plants with enhanced traits, such as disease resistance, abiotic stress tolerance, wood yield and quality, and fruit characteristics [4,5,6].

However, genetic transformation of woody plants is fraught with challenges due to their obscure genomes, inherent recalcitrance to in vitro manipulation, and pronounced variability in transformation efficiency across species and even among genotypes within the same species [7,8,9,10]. The process of plant transformation typically involves two critical steps: the delivery of genetic material into plant cells and the subsequent plant regeneration from those transformed cells [11]. To date, multiple strategies have been explored for delivering DNA into woody plants, such as methods using *Agrobacterium tumefaciens* [12,13], *Rhizobium rhizogenes* [14], particle bombardment [15], and nanoparticles [16]. Each of these methods has distinct advantages, yet none reliably enables high-efficiency, genotype-independent transformation in woody plants. Moreover, plant regeneration from transformed cells remains a major obstacle for most woody plants. This field is now undergoing a paradigm shift. The historical reliance on labor-intensive, genotype-specific, tissue culture-based transformation protocols is being superseded by the emergence of engineered, precise, and increasingly universal strategies. This transition is catalyzed by transformative advances in synthetic biology, multi-omics technologies, and innovative biomolecular delivery platforms. Landmark achievements, such as the successful generation of transgene-free edited poplar [17] and the demonstration that developmental regulators can substantially enhance transformation efficiency [18], highlight the shift in next-generation transformation strategies from conceptual feasibility to practical implementation. In this review, we evaluate this evolving landscape, delineating the current state of the field, the principal challenges that remain, and the prospects for future innovation in woody plant transformation.

## 2. Methodological Paradigms in Woody Plant Transformation

Woody plant genetic transformation employs a range of methods that can be broadly categorized into two strategic paradigms. The first involves the modernization of foundational, tissue culture-based techniques, aiming to “fix the system” by making it more efficient and predictable. The second, driven by the persistent difficulties of the first, seeks to “change the system” entirely by developing non-tissue culture-based strategies that bypass the bottlenecks of tissue culture-based methods.

### 2.1. Tissue Culture-Based Platforms for Woody Plant Transformation

**Figure 1:**
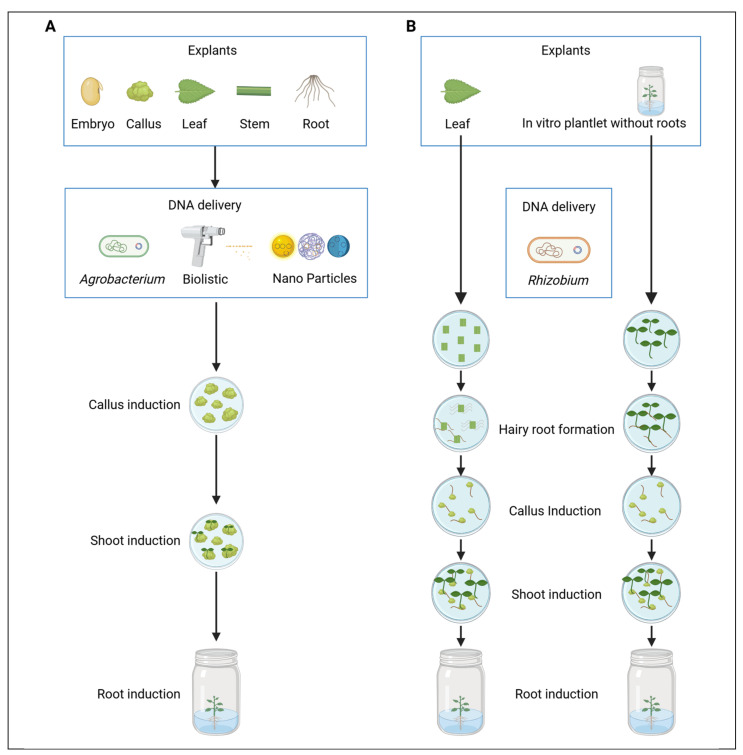
Tissue culture-based transformation methods for woody plant transformation. (**A**) *Agrobacterium*-mediated transformation. (**B**) *Rhizobium*-mediated transformation. Redrawn from [48,49,50,51]. Created with https://BioRender.com.

Tissue culture-based plant transformation involves introducing genetic material, such as genes of interest, into in vitro-grown explants (plant cells or tissues) via bacterial vectors (e.g., *Agrobacterium tumefaciens*, *Rhizobium rhizogenes*), particle bombardment, or nanoparticle-mediated DNA delivery, and then regenerate them into whole plants with desired traits, either directly or indirectly through callus formation, which may originate from the explants themselves or from hairy roots induced by *R. rhizogenes* (Figure 1). In this section, we discuss advances in DNA delivery and plant regeneration relevant to tissue culture-based transformation in woody plants.

#### 2.1.1. Advances in DNA Delivery for Tissue Culture-Based Transformation in Woody Plants

*A. tumefaciens*-mediated transformation remains the most widely used approach for transforming woody plants. The bacterium’s tumor-inducing (Ti) plasmid facilitates stable integration of foreign DNA in the plant genome [19]. The Ti plasmid contains the virulence (*Vir*) genes encoding proteins required for T-DNA transfer, which are usually expressed at low levels, but acetosyringone can trigger a strong increase in their expression via the VirA/VirG two-component regulatory system, facilitating DNA transfer from *Agrobacterium* to plant cells [20,21,22]. Various *Agrobacterium* strains (often carrying binary vectors or even “super binary” and ternary vector systems) have been developed to improve transformation efficiency [23]. *A. tumefaciens* has proven effective in transforming leaf, petiole, stem (internode), node, and root explants in *Populus* [12,24]; shoots and hypocotyl explants of *Eucalyptus* [10,18]; and hypocotyl and leaf explants of *Prunus* [10,18].

Hairy root transformation via *Rhizobium rhizogenes* has been developed as an alternative to *A. tumefaciens*-mediated transformation [25]. *R. rhizogenes* is a Gram-negative soil bacterium that infects various dicotyledonous plants (dicots). It typically carries two T-DNA regions (TL-DNA and TR-DNA) on its Ri plasmid that can be independently transferred into host plant genome. The TL-DNA contains a family of 18 genes [26], with four (i.e., *rolA*, *rolB*, *rolC*, *rolD*) of them responsible for hairy root induction [27], while the TR-DNA contains genes for the biosynthesis of auxin [28] and agropine [29].

The primary advantage of *Agrobacterium*/*Rhizobium*-mediated transformation is stable integration of usually a single or a few transgene copies, supporting more predictable gene expression. This method of transformation has been successfully utilized in *Malus domestica*, *Actinidia chinensis* [30], *Citrus* [14], *Populus* [31], and *Eucalyptus* [32] using leaf or in vitro-grown plants as a explants. However, this approach is constrained by a limited host range and its dependence on slow, genotype-specific tissue culture protocols.

Particle bombardment (biolistic) is a physical method to directly introduce foreign DNA into plant cells using high velocity microprojectiles. Once inside the cell, the DNA can separate from the microprojectiles and may undergo transient expression or stable genomic integration [33]. This approach has been used to transform various woody plant species, such as *Betula pendula* [34], peach embryo calli [35], *Tripterygium wilfordii*, *Castanea dentata* [36], *Carica papaya* [37], *Larix gmelinii* [38], *Picea abies* [39], and *Pinus radiata* [40]. Particle bombardment offers several advantages over *Agrobacterium*-mediated transformation, including applicability to recalcitrant species and diverse tissues, efficient co-transformation of multiple genes or genomes (including organelles), delivery of DNA-free biomolecules, insertion of large DNA fragments, and the use of minimal expression cassettes that reduce unwanted vector integration [41]. However, this approach is employed less frequently than *Agrobacterium*-mediated methods in plant transformation because of several limitations, including high equipment and consumable costs, complex integration patterns, low transformation efficiency, high chimerism, poor genetic stability, and the frequent occurrence of multiple transgene copies, which can result in abnormal expression and co-suppression [42].

Nanoparticle-mediated gene delivery is a new emerging technology in the field of genome engineering. Recently, different types of nanoparticles have been tested including carbon-based nanoparticles, metal-based nanoparticles, magnetic nanoparticles, and cell-penetrating peptides [43]. Magnetic nanoparticles have been used to introduce exogenous DNA into pollen with stable integration of targeted transgenes in *Gossypium hirsutum* Linn. [44]. Advantages include (1) increased stability of genetic transformation by protecting genetic material and minimizing tissue damage [45], (2) allowing for direct cellular delivery of genetic material [46] and (3) enhancing transformation efficiency for both temporal (transient) and permanent (stable) genetic modifications [47]. The disadvantages include phytotoxicity of nanoparticles in the transformation products [16] and cytotoxicity in plant cells because of the accumulation of high-density charged polymer [47].

#### 2.1.2. Advances in Plant Regeneration for Tissue Culture-Based Transformation in Woody Plants

Improving plant regeneration for tissue culture-based transformation in woody plants remains one of the major bottlenecks in developing stable transgenics. The use of plant hormones, developmental regulators, and small molecules and related compounds can significantly enhance regeneration efficiency, paving the way for successful genetic improvement of these economically and ecologically important plants.

Recent progress in this area highlights how the fine-tuning of hormonal regimes can help overcome species-specific barriers in *A. tumefaciens*-mediated transformation. For example, exogenous hormone supplementation to tissue culture media significantly enhances regeneration efficiency in multiple woody plant species, such as *Populus tremula* [52], *P. tremula* × *P. alba* [53], *Eucalyptus*, [54], and *Prunus* [55].

Building on hormonal strategies, manipulating key developmental regulators has significantly enhanced regeneration in woody plants. The *BABY BOOM (BBM)* gene, a well-known developmental regulator [56,57,58], has been used to improve transformation efficiency in several woody plant species. For instance, *MdBBM1* pre-transformation enhanced apple re-transformation [18]. Also, *LhBBM* overexpression, under both constitutive and somatic embryogenesis-specific promoters, increased embryonic callus induction and transformation in *Liriodendron* hybrids [59]. Furthermore, *TcBBM* overexpression triggered somatic embryogenesis in *Theobroma cacao* without external hormones [60]. Beyond *BBM*, several other developmental regulator genes have been employed to improve transformation efficiency in woody plants. For example, the overexpression of *WOX* or *KNOX* homeobox genes improved somatic embryogenesis in *Pinus pinaster*, though rooting remains a challenge [61,62,63,64,65]. Also, the efficiency of citrus transformation was improved by overexpressing maize *kn1* [66], *GRF4*-*GIF1* [67]. In addition, ectopic expression of *CsL1L* driven by the 35S promoter induced embryo-like structures in citrus, indicating a role in cellular reprogramming [68].

Beyond plant hormones and developmental regulators, small molecular and related compounds are gaining attention for enhancing regeneration in woody plants. Genetic transformation in conifers is hindered by technical and regulatory barriers, but combining small molecules (e.g., GSK-3β) [69], REDOX regulators, epigenetic agents, and signaling peptides with growth regulators shows promise for improving somatic embryogenesis [70]. For instance, it was reported that 5-azacytidine treatment, which acts as a DNA methyltransferase inhibitor to cause DNA hypomethylation, increased fresh mass and embryo maturation in *P. pinaster* [71]. Also, treatment with trichostatin A (TSA), a potent inhibitor of histone deacetylase (HDAC) enzymes, maintained embryogenic potential despite partial germination delays in *Picea abies* [72] and *P. sylvestris* [73]. Additionally, metal nanoparticles (MNPs) improve organogenesis and eliminate microbial contamination when used as elicitors or stress agents [74].

### 2.2. The Rise in Non-Tissue Culture-Based Platforms for Woody Plant Transformation

**Figure 2:**
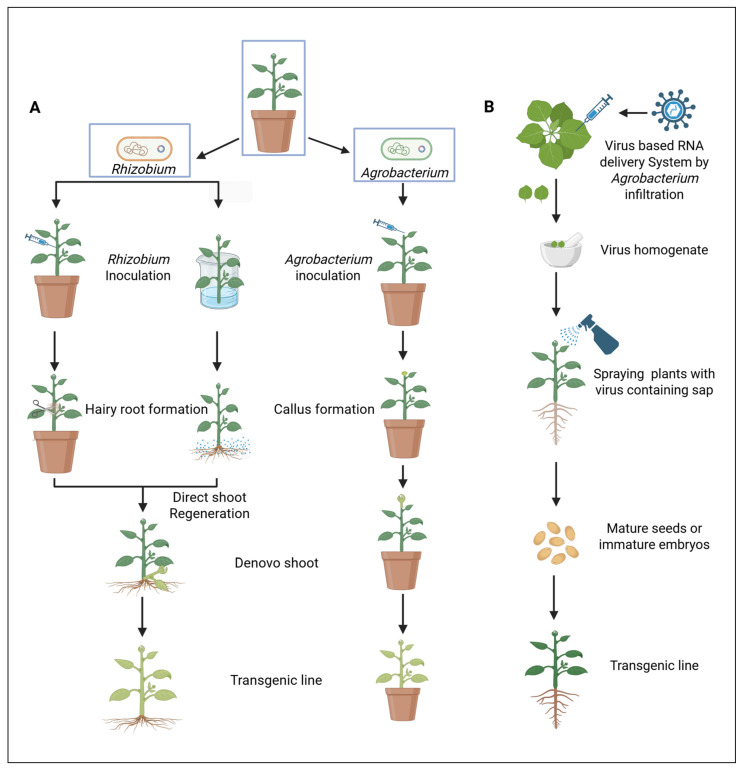
Non-tissue culture-based transformation methods for woody plant transformation. (**A**) In planta transformation mediated by *Rhizobium rhizogenes* and *Agrobacterium tumefaciens*. (**B**) Virus-mediated transformation. Redrawn from [14,49,51,75,89]. Created with https://BioRender.com.

The development of efficient transformation systems for woody plants remains a significant challenge, largely because existing protocols rely heavily on tissue culture. Such methods are often labor-intensive, time-consuming, and strongly genotype dependent. Many woody species are recalcitrant to regeneration, resulting in low transformation efficiency and constraining opportunities for genetic improvement. To address these limitations, non-tissue culture-based transformation methods (Figure 2) have emerged as promising alternatives. By bypassing in vitro regeneration, these approaches reduce costs, minimize somaclonal variation, and preserve genetic integrity. They also enable new possibilities for direct genome editing and transgene-free applications—particularly valuable for perennial species with long generation times. Nevertheless, even without the reliance on tissue culture, non-tissue culture-based transformation still requires efficient DNA delivery and plant regeneration. In the following section, we review recent advances in these two critical components as they relate to non-tissue culture-based transformation in woody plants.

#### 2.2.1. Advances in DNA Delivery for Non-Tissue Culture-Based Transformation in Woody Plants

In planta transformation techniques introduce genetic material directly into plant tissues—typically meristems, embryos, or reproductive structures—without the need for callus induction or regeneration in vitro. This approach has emerged as a valuable alternative to conventional tissue culture-dependent transformation methods, particularly for species that are recalcitrant to regeneration. The major advantages of in planta transformation are (1) avoiding complex tissue culture, (2) reducing cost, labor, and time compared to traditional method, (3) minimizing somaclonal variation and preserving genotype integrity, and (4) increasing the potential for transgene-free genome editing via transient expression systems. Several innovative in planta methods have been developed and tested in woody species. For instance, the Cut Bud Dipping method was successfully applied in citrus by excising apical buds and dipping them in *Agrobacterium* suspension [75], while in poplar, transformation was achieved by injecting *Agrobacterium* into closed floral buds during early winter [76]. The Fast-TrACC (fast-treated *Agrobacterium* co-culture at the seedling stage) approach has produced bioluminescent shoots in grapes [77]. A tissue culture-free hairy root transformation system was established in *Camellia sinensis* [78] and poplar [79] using leaf explants, and diverse citrus cultivars such as grapefruit—Rio Red (*Citrus paradisi*), sweet orange—Valencia (*Citrus sinensis*), rough lemon (*Citrus jambhiri*), carrizo (*Citrus poncirus*), and citron (*Citrus medica*) were successfully transformed by immersing epicotyls in *R. rhizogenes* suspension [14]. Similarly, cut-dip budding with *R. rhizogenes* enabled heritable transformation in *Ailanthus altissima*, *Aralia elata*, and *Clerodendrum chinense* [80]. Another approach, meristematic bulk induction, generated transgenic shoots from the shoot apices of proliferating shoots that were transformed using *Argobacterium* deliver GFP into six grape cultivars, achieving transformation efficiencies comparable to those obtained with conventional techniques [81]. Despite these advances, efficiencies remain low, and outcomes are highly dependent on species and developmental stage. Overall, such in planta methods represent a promising frontier in woody plant transformation, though they are not yet reliable, routine tools.

Viral vector-based transformation is another emerging technology that allows plant transformation that is free from foreign DNA [82]. It uses RNA guides instead of DNA, rendering the transformed material to be transgene free. Virus-induced gene silence has been widely used to study gene function in plants. This approach enables transient or, in some cases, stable expression of target genes without the need for complex tissue culture steps, making it especially useful for functional genomics studies and high-throughput screening. Viral vectors such as tobacco rattle virus (TRV), potato virus X (PVX), and foxtail mosaic virus (FoMV) have been commonly employed for virus-induced gene silencing (VIGS) and gene expression in a wide range of plant species [83,84]. Innovations in synthetic biology and CRISPR-based tools have expanded the utility of viral vectors for precise genome editing and multiplex gene regulation in planta [85]. Despite some limitations in cargo size and host range, viral vectors represent a versatile and powerful platform for plant transformation. Engineered Telosma mosaic virus (TelMV-VIGS) has been used to silence the phytone desaturase (PDS) gene in passion fruit by rub inoculation on the cotyledons [86]. *Citrus leaf blotch virus* (CLBV)-based viral vector was used to develop early flowering citrus [87]. While excellent for research and functional genomics, they are less suited for creating stable commercial crop lines.

#### 2.2.2. Advances in Plant Regeneration for Non-Tissue Culture-Based Transformation in Woody Plants

Non-tissue culture-based transformation methods bypass the tissue culture bottleneck. Improving the recovery and regeneration of transformed plants through these techniques is a critical frontier, with success hinging on novel approaches that directly target the actively growing plant parts. Unlike tissue culture, where regeneration involves inducing whole plants from undifferentiated cells, “regeneration” in a non-tissue culture context refers to the successful development of a genetically modified germline cell into a viable, non-chimeric transgenic plant. The primary challenge lies in the low frequency of transformation and the difficulty of ensuring that genetic modification is incorporated into the reproductive cells that will give rise to the next generation. To overcome these limitations, recent efforts have turned toward molecular strategies that enhance the intrinsic regenerative capacity of plant tissues, particularly through the modulation of key developmental regulators. For example, developmental regulators *WUSCHEL* (*WUS, ZmWUS2*) and *SHOOT MERISTEMLESS* (*STM*, *AtSTM*) and de novo meristem induction factors (*LAS*, *RAX1*, *EXB1*, *ROX*, and *ARR1*) were used to improve regeneration efficiency for in planta genome-editing system for citrus by cutting the stem of 4-month-old seedlings and applying a mixture of *Agrobacterium*, resulting in regeneration efficiency up to 75% [88]. Kelly et al. [88] also reported *IPT*, *GRF4-GIF1*, and *PLT5* improved transformation and regeneration efficiency in four different citrus cultivars using in planta genome-editing system.

## 3. Species-Specific Advances in Woody Plant Transformation

Transformation technology in woody plants is best illustrated through targeted case studies that showcase both significant advancements and persistent obstacles. While both somatic embryogenesis (SE) and organogenesis have contributed to progress in this field, organogenesis stands out for its ability to rapidly produce large numbers of plants and its relative independence from genotype constraints. In contrast, SE remains hindered by multiple challenges such as low initiation frequency, abnormal embryo development, and strong genotype sensitivity [70]. Despite its own complexities, organogenesis continues to be the more widely adopted method for in vitro regeneration of woody species. The following sections therefore focus on recent developments across seven (*Citrus*, *Eucalyptus*, *Malus*, *Pinus*, *Populus*, *Prunus*, and *Salix*) selected for their economic or ecological importance and the active research within each group, where organogenesis has been the primary regeneration strategy (Table 1).

### 3.1. Citrus spp.

*A. tumefaciens* is the most used method of transformation for genetic engineering in citrus, with efficiency ranging up to 45% using epicotyl as explants [97]. An in planta transformation methodology employing various *Agrobacterium* application techniques—such as blunt cut and apical cut dip—achieved transformation efficiencies of up to 4.17% across multiple citrus species [75]. *R. rhizogenes* have been recently used for citrus transformation by hairy root induction with efficiency around 57% in 2–8 weeks for gene function analysis. As noted above, [98] transformed *C. medica*, *C. limon*, and citrange ‘Carrizo’ using *R. rhizogenes* (K599), and hairy roots were induced after 2–4 weeks with efficiency up to 57% in *C. medica*. Various citrus species were transformed using *R. rhizogenes* with transformation efficiencies of 28–75% with successful regeneration of transgenic lines [14] in about 6 months duration. Transgenic *Citrus sinensis* × *Poncirus trifoliata* shoots were developed in 1–1.5 months using biolistic transformation [15]. Mature tissue transformation has also been carried out for citrus transformation using mature stem as it bypasses the juvenile stage, reducing the time and costs involved in evaluating transgenic new traits [99,100]. The problem though with mature transformation is very low regeneration efficiency [101]. So, the success of citrus transformation varies significantly depending on the species and genotype. Although *R. rhizogenes*-mediated transformation looks promising for citrus, it is still necessary to improve the efficiency of shoot regeneration from transformed hairy roots.

### 3.2. Eucalyptus spp.

*Eucalyptus globulus* stable transformation was carried out by a biolistic method using zygotic embryos [102]. Thanananta et al. [91] transformed *Eucalyptus camaldulensis × E. tereticornis* using peeled nodal-stem explants from in vitro propagated plantlets using *A. tumefaciens* with an efficiency of 24%. A hybrid *Eucalyptus* cultivar (*Eucalyptus urophylla* × *E. grandis*) was transformed by *Agrobacterium* to introduce *g10-epsps* gene, driven by the 35 S promoter with an efficiency of 4.0%, which took more than 14 weeks to generate transgenic plants [90]. Hairy root induction with *R. rhizogenes* has been successfully demonstrated in *E. grandis* using the following four different inoculation methods: (1) hypocotyl stabbing in a seedling, (2) cutting and inoculating the base of the hypocotyl of 14-day-old seedlings, (3) cutting and inoculating the radicle apex of three-day-old germinating seeds, and (4) stem stabbing of an in vitro clonal line with three different strains of *R. rhizogenes* (i.e., AR4S, A4 and ARqua1), of which hypocotyl stabbing with AR4S strain resulted in a higher transformation efficiency of 62.2% for hairy root induction only [32]. The genetic improvement of *Eucalyptus* is severely hampered by the lack of an effective and complete regeneration protocol. While methods exist for the initial “transformation” step, the subsequent and crucial “regeneration” phase is largely unsuccessful.

### 3.3. Malus Domestica

*A. tumefaciens* have been the most used method of transformation for genetic engineering in apple. Most of the transformation is carried out from leaf explants. Young expanding leaves from 4-week-old cultures have been used as explants for transformation [18,103]. *R. rhizogenes* has also recently been used to develop a hairy root transformation protocol using leaves as explants to identify root development-related genes [30]. The leaves were co-cultured with *R. rhizogenes* for 3 days, followed by 10 days of culture in rooting medium to induce transgenic hairy root formation, which were further grown for 10 days. The GFP-positive explants with roots were used for shoot induction with a regeneration efficiency of 3.3% in 14 weeks, which further improved to 20.6% in 9 weeks with the use of *MdWOX5*. Apple transformation efficiency also varies with cultivar, where efficiency in ‘JM2’ ranges from 0.4 to 3.1% [104] and cultivar ‘M.26’ ranges from 22 to 25.4%. Improvements in apple transformation have been slow; regenerating whole plants from modified cells currently has low success rates. Developing more efficient transformation methods is crucial for the overall improvement of commercial apple cultivars. Incorporating transcription factors, such as *MdBBM*, in the pre-transformation stage of *A. tumefaciens*-mediated transformation markedly enhanced transformation efficiency, raising it from 3% to 30% [18]. Although these improvements have expanded the options for apple transformation, the lack of a standardized, rapid, and reliable method across genotypes remains a major challenge for achieving higher efficiency.

### 3.4. Pinus spp.

*A. tumefaciens* has been successfully used for genetic engineering in *Pinus*. Liu et al. [105] transiently transformed *Pinus tabuliformis* using *A. tumefaciens* (LB404) with a transformation efficiency of 70.1% without successful regeneration. *Pinus massoniana* zygotic embryos have been successfully transformed using *Agrobacterium tumefaciens* strain EHA105, achieving transformation efficiencies ranging from 6% to 59% depending on the specific combinations of *Agrobacterium*) and acetosyringone treatments, with an optimal condition combining an *Agrobacterium* density of OD_600_ = 0.5, cold treatment of the bacterial suspension at 4 °C for approximately five hours, a five-hour inoculation period, and supplementation of the co-cultivation medium with 100 µM acetosyringone [92]. *R. rhizogenes* has also been used to transform *Pinus contorta* with the efficiency of 20–50% for hairy root induction [106]. Despite the success, *Pinus* transformation faces a major obstacle of successful regeneration of transgenic plants as it is genotype-dependent and only a few cases of success have been reported. Further research is needed to achieve both effective transformation and the regeneration of stable transgenic lines for practical benefits.

### 3.5. Populus spp.

*Populus* is the model woody plant for genomics research. *A. tumefaciens* has been the most popular transformation method for *Populus*. There are challenges in transformation among species and genotypes of *Populus*. *P. tremula* × *P. alba* ‘INRA 717-1B4’ has been the most successful genotype for transformation for which leaf, petiole, and stem can be used as explants for transformation with good regeneration capacities of 15–25% [12]. *Populus deltoides* ‘WV94’ exhibited a low transformation efficiency of approximately 8.7% when tissue-cultured leaf explants were transformed using *A. tumefaciens* [13]. *P. trichocarpa* ‘Nisqually-1’ showed a regeneration efficiency of 13% from young stem explants transformed using *A. tumefaciens* [93]. Sulis et al. [94] reported biallelic editing efficiency of 86.1% for *PtrPAL4* and *PtrPAL5* to 9.3% for *PtrCCoAOMT2* for *P. trichocarpa* ‘Nisqually-1’ using *A. tumefaciens* for transformation. Even though *A. tumefaciens*-mediated transformation has been a prevalent method for *Populus* transformation, low efficiency and long regeneration time remain as barriers. Hairy root induction using *R. rhizogenes,* another method of transformation, is gaining popularity for *Populus* transformation. Reference [31] transformed ‘INRA 717-1B4’ using *R. rhizogenes* to produce secondary metabolites. Strains ATCC 15834, A4 (ATCC 31798), LBA 9402 (NCPPB 1855), and MSU 440 were tested for hairy root induction. MSU 440 was the most efficient in inducing hairy roots with an efficiency of 80% [31]. *Rhizobium rhizogenes*-mediated hairy root induction can be an alternative to existing transformation using *Agrobacterium*, if regeneration of transgenic plants can be successfully carried out. In planta transformation can be another alternative for poplar transformation to solve the genotype dependency issue. The uniformity of the method of transformation needs research to improve the efficacy of this method.

### 3.6. Prunus spp.

As with most woody species, *A. tumefaciens* has been the method of choice for transforming *Prunus.* Prieto et al. [95] transformed different species of *Prunus* using strain GV3101. *Prunus persica* embryo and cotyledons explants resulted in regeneration efficiencies of 2% and 0.5–1%, respectively, while *Prunus salicina*, using epicotyl and hypocotyl explants, resulted in regeneration efficiencies of 17% and 3%, respectively, and *Prunus avium*, using hypocotyls, displayed a regeneration efficiency of 4%. Ref. [107] reported an efficient root transgenic system using *R. rhizogenes* using leaf, hypocotyl, and shoot to induce hairy root only with transformation efficiency of over 50% to validate the function of an anthocyanin-related regulatory gene *PpMYB10.1* in transgenic hairy roots. *Agrobacterium*-mediated transformation protocol was established for in vitro leaf explants derived from elite, mature *Prunus serotina* tree, yielding a transformation efficiency of 1.2% [108]. The transformation process for *Prunus* species is lengthy, often requiring up to 12 months to produce transgenic lines. The efficiency of regeneration in *Prunus* is low and merits further research to enhance the efficiency of this process.

## 4. Challenges and Potential Solutions in Woody Plant Transformation

**Figure 3:**
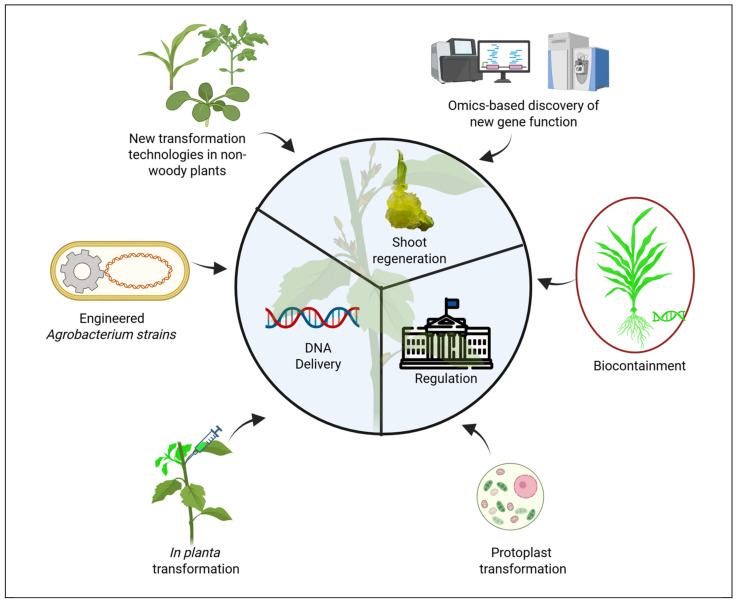
Potential solutions to the major challenges in woody plant transformation. Created with https://BioRender.com.

Across woody plant transformation platforms, three persistent challenges prevail: achieving efficient gene delivery to plant cells, reliably regenerating whole, non-chimeric plants, and navigating regulatory pathways toward commercialization. These hurdles can be addressed by adapting innovations from non-woody plant systems, elucidating the molecular basis of recalcitrance in woody species, and engaging with the complex regulatory and societal frameworks required to translate laboratory advances into real-world applications, as summarized in Figure 3.

### 4.1. Adapting Innovations from Non-Woody Plant Systems

Non-woody model systems have driven the development of tools and concepts that have radically improved transformation through enhancing both DNA delivery and plant regeneration. For example, the efficiency of DNA-delivery mediated by *Agrobacterium* was significantly enhanced by engineering *A. tumefaciens* with type III secretion systems (T3SS) to deliver defense-suppressing effectors, resulting in an improvement of transformation efficiency up to 250–400% in wheat, alfalfa, and switchgrass [109]. Also, an *Agrobacterium* ternary vector system, which includes a T-DNA binary vector and a compatible helper plasmid, has proven effective in improving transformation efficiency in maize [110]. These new advancements in non-woody species highlight the potential of engineering *Agrobacterium* strains and T-DNA vectors to enhance DNA delivery in *Agrobacterium*-mediated transformation of woody plants.

In addition to new advances in DNA delivery, exciting new achievements have recently been made to improve shoot regeneration in non-woody plants. For example, in tomato, transcription factors such as PORK1—a receptor of REF1—promote regeneration by activating the master regulator WIND1 through REF1–PORK1-mediated signaling, in which REF1 functions as a local wound signal that triggers plant regeneration [111]. HY5-mediated light signaling promotes shoot regeneration in pre-transformed *Arabidopsis thaliana* with a loss-of-function mutation in COP1, which is the upstream regulator repressing HY5 activity by targeting it for degradation [112]. Additional examples include overexpression of maize *GOLDEN2* (a member of the GARP transcription factor superfamily), which enhances regeneration in rice by promoting chloroplast development [113]. Similarly, *LAX1* overexpression markedly boosts regeneration efficiency in wheat, maize, and soybean, indicating that this approach can improve regeneration in both monocot and dicot crops [114]. Complementing these transcription factor-based strategies, recent studies have also explored epigenetic regulation as a powerful means to modulate plant regenerative competence. Epigenetic inhibitors (EPIs) have been applied in plant regeneration research, where they alter DNA methylation or histone modifications, resulting in a decrease in morphogenic regulator activity [70]. For instance, trichostatin regulates histone acetylation, which enhances the expression level of the *LEC1*, *LEC2*, *FUS3*, and *MYB118* genes positively in *Arabidopsis* [115]. The combined use of sodium butyrate and trichostatin enhances histone acetylation, upregulating *BBM* and *SERK2* in grape [116] and improving embryogenic responses in wheat [117]. Building upon these molecular and epigenetic innovations, advances in transformation methods now aim to overcome genotype-dependent barriers to regeneration. In addition, selecting transgenic events is another challenge. In particular, natural tolerance to antibiotics (e.g., kanamycin resistance in chestnut [118]) and chimerism [119] can complicate effective identification of true transformants. These challenges can be addressed by using visual markers (GUS [120], GFP [121], DsRed [122], RUBY [123] in combination with traditional selectable marker genes, such as herbicide- or antibiotic-resistance genes.

### 4.2. Elucidating the Molecular Basis of Recalcitrance in Woody Species

While recent advances in transformation technologies for non-woody plants offer valuable insights, they fall short of resolving the persistent challenges in woody species due to fundamental biological differences and the species-specific nature of plant transformation [124]. A comprehensive understanding of the molecular mechanisms underlying recalcitrance in woody plants is essential to bridge this gap. Woody plants are difficult to transform due to complex molecular factors, including genetic variation, hormone imbalances, and epigenetic regulation. The obstacles to transformation arise as soon as DNA is delivered, since wounding in woody tissues often triggers intense defense responses that quickly hinder *Agrobacterium* infection and compromise explant survival. Plant responds to *Agrobacterium* via three-layered immunity (pathogen signal perception and innate plant immunity, MAPK signaling, and defense responsive genes) that determines its susceptibility or resistance to *Agrobacterium* infection [125,126]. Plant defense includes release of reactive oxygen species (ROS) and phytohormones like salicylic acid and jasmonic acid [127]. In barley, ROS accumulation during *Agrobacterium* infection was similar among genotypes tested, but comparative transcriptomics analysis revealed upregulation of seven genes related to plant hormone signal transduction and DNA replication only in compatible genotypes [128]. Salicylic acid interferes with *Agrobacterium tumefaciens* infection by suppressing *vir* gene expression, limiting bacterial growth, reducing attachment to plant cells, and weakening overall virulence [129,130]. To address this issue, super-infective ternary vector systems have been created that incorporate salicylic acid-degrading enzymes along with GABA and ethylene-degrading activities, enabling more efficient crop transformation by suppressing plant defense responses against *Agrobacterium* [131].

Different omics approaches have been employed for studying the molecular basis of transformation and regeneration, which have revolutionized the discovery of developmental and morphogenic regulators that are involved during transformation in both woody and non-woody species. Recent advances in single-cell and spatial transcriptomics have provided unprecedented insights into cell fate determination and reprogramming during plant regeneration. For instance, single-cell RNA-seq identified two transcription factors, *WUS* and *DRN*, whose ectopic activation promotes protoplast regeneration in *Arabidopsis* [132]. Similarly, Quartz-Seq2-based single-cell transcriptomics revealed that *WOX13* plays a key role in determining cellular identity within callus cell populations and suppressing meristem regulators in *Arabidopsis* [133]. In *Gossypium* (cotton), single-cell transcriptomics uncovered the regulatory networks governing somatic embryogenesis and provided new perspectives on cell fate transition and reprogramming during regeneration [134]. Spatial transcriptomics in tomato further mapped diverse callus cell types and identified chlorenchyma cells as critical contributors to shoot regeneration, offering single-cell-level insights into regeneration mechanisms in crop callus [135]. Beyond transcriptomics, integrative multi-omics and network analyses are revealing the complex hormonal and transcriptional regulation underlying transformation and regeneration. RNA-seq, ATAC-seq, and CUT&Tag analyses of auxin-induced embryonic callus revealed transcriptional regulatory networks, identifying *TaDOF5.6* and *TaDOF3.4* as candidate factors that could enhance transformation efficiency in wheat [136]. Similarly, genome-wide association studies (GWAS) and network analyses of in vitro transformation in *P. trichocarpa* highlighted key roles of diverse phytohormone pathways and cross talk during transformation and regeneration [137]. In woody plants, genomics approaches are being increasingly used to identify novel regulators that influence regeneration competence. Genomics approaches have also been used in woody plants to identify novel genes involved in plant regeneration. Tuskan et al. [138] employed GWAS to identify eight transcription factors regulating callus formation in *Populus*, while [139] further identified several genes associated with stem and root regeneration. Collectively, these findings underscore the power of omics-driven discovery in revealing the molecular mechanisms of regeneration and transformation. Harnessing these insights will be crucial for enhancing transformation efficiency and overcoming recalcitrance in woody plant genotypes.

### 4.3. Challenges in Regulatory Approval and Commercialization

Regulatory approval of engineered woody plants faces significant uncertainty due to inconsistent global frameworks. While regions such as the United States, parts of Africa, Asia, and Latin America have adopted flexible approaches to commercialization of genetically modified organisms (GMOs) [140], other regions, such as the European union [141] and South Africa [142], maintain strict regulations that increase costs, delay approvals, and influence research and development decisions [143,144]. The absence of harmonized international guidelines confounds these challenges, particularly for woody plants, where long life cycles further extend testing timelines. Additional barriers include public resistance fueled by misconceptions, concerns over gene flow to wild relatives, and intellectual property constraints that increase licensing costs and limit access.

To address these issues, internationally aligned guidelines distinguishing transgenic from non-transgenic products should be developed. Molecular markers and multi-omics tools could help accelerate regulatory testing. Public awareness campaigns highlighting DNA-free editing approaches and their benefits for sustainable crop improvement are essential. DNA-free editing includes approaches like Cisgenesis, which refers to the genetic modification of a plant using a native gene sourced from a sexually compatible, crossable species [145], and may provide some relief. Protoplast transformation which does not include introduction of foreign DNA, which is a key step in DNA-free gene editing using CRISPR/Cas9 RNPs [146], may also ease public concern. There are some edited crops that have already been approved for sale like the gene-edited CRISPR mushroom developed by [147] and *Camelina sativa*, or false flax, with enhanced omega-3 oil developed by [148]. Genome-edited Waxy Corn Product developed by Corteva Agriscience by using CRISPR-Cas9 technology is another example [149].

Ensuring biosafety through gene containment strategies and long-term ecological monitoring will also be critical. Physical methods like isolation distances and separation crops [150] and reproductive isolation [151] can be implemented to contain gene flow. Molecular approaches like maternal inheritance, male sterility, seed sterility, cleistogamy and apomixis, genome incompatibility, temporal and tissue-specific control, and transgenic mitigation can also be utilized to address biosafety issues [152,153]. Encouragingly, regulations in many countries are gradually adapting to new technologies [154,155] and current trends suggest cautious optimism that gene editing will achieve broader public acceptance and play a transformative role in global food security and environmental sustainability [156].

## 5. Conclusions and Future Opportunities

Genetic transformation holds great promise for accelerating the improvement of woody species. Despite this potential, practical implementation of transformation technologies continues to face major hurdles. *Agrobacterium*-mediated transformation remains the predominant approach for woody plants, though its success is highly dependent on species, genotype, tissue type, and specific protocols. *R. rhizogenes* presents a faster and more efficient alternative, particularly useful for recalcitrant species or root-focused studies. However, challenges such as poor regeneration capacity, low transformation efficiency, and extended timelines continue to limit its broader application. Addressing these limitations will require optimization of culture conditions, exploration of novel regeneration pathways, use of developmental regulators, early non-destructive selection markers, and advanced molecular techniques. A major barrier to progress remains the lack of a reliable, efficient, and broadly applicable transformation and regeneration system for the most commercially important woody plants. Overcoming this will demand a multi-faceted approach—improving existing methods, converting emerging technologies into practical applications, and deepening our understanding of the molecular processes that make woody species resistant to transformation and regeneration. Future efforts should prioritize the development of more efficient approaches such as in planta transformation, DNA-free genome editing, and nanoparticle-based delivery methods. These innovations could make transformation faster, more efficient, and more universally applicable. Continued investigation into the molecular foundations of regeneration and transformation will be essential to establish robust protocols that can be widely used across diverse woody plant species and genotypes.

## Figures and Tables

**Table 1:** Examples of stable transformation in woody plants. AT, *Agrobacterium tumifaciens*; RR, *Rhizobium rhizogenes*.

Species	Explant	DNA Delivery	Organ Regeneration	Transformation Time	Transformation Rate (%)	References
*Citrus* × *poncirus*	Seedlings	RR	Hairy root induction	6 months	28–75%	[14]
*Eucalyptus urophylla* × *E. grandis DH3229*	Leaves	AT (Strain EHA105)	Callus-mediated organogenesis	>14 weeks	4.07%	[90]
*E. camaldulensis* × *E. tereticornis*	Peeled nodal stem	AT (Strain EHA105)	Direct regeneration	>8 weeks	~24%	[91]
*Malus domestica*	Leaves	RR (Strain K599)	Hairy root induction	14 weeks	3.3–20.6%	[30]
*Malus domestica*	Leaves	AT (Strain LBA4404)	Direct regeneration	4 months	3–30%	[18]
*Pinus massoniana* Lamb.	Embryo	AT (Strain EHA105)	Callus-mediated organogenesis	15 weeks	6–59.75%	[92]
*Populus tremula* × *P. alba clone INRA 717-1B4*	Leaves, petioles, roots	AT (Strain GV3101)	Callus-mediated organogenesis,	>13 weeks	15–25%	[12]
*P. deltoides*	Petioles and base of binary vein	AT (Strain EHA105)	Direct regeneration	3–4 months	8.7%	[13]
*P. trichocarpa*	Internodal stem	AT (Strain C58)	Callus-mediated organogenesis	5–8 months	13%	[93,94]
*Prunus persica*	Embryo, cotyledon	AT	Callus-mediated organogenesis	8–12 months	2%	[95]
*Prunus avium*	Epicotyl	AT (Strain GV3101)	Callus-mediated organogenesis	6–8 months	4%	[95]
*Salix matsudana*	Embryogenic shoots	AT (Strain LBA4404)	Callus-mediated organogenesis	~5 months	7.2%	[96]

## Abbreviations

The following abbreviations are used in this manuscript:

DNA	Deoxyribonucleic acid
RNA	Ribonucleic acid
CRISPR	Clustered regularly interspaced short palindromic repeats
TALENS	Transcription activator like effector nuclease
RNPs	Ribonucleoproteins
MS	Murashige and Skoog medium
WPM	Woody plant basal medium
NAA	Naphthalene acetic acid
IAA	Indole-3-acetic acid
2.4-D	2,4-Dichlorophenoxyacetic acid
BA	6-Benzylaminopurine
TDZ	Thidiazuron
GUS	β-glucuronidase
GFP	Green fluorescence protein
